# Global Transcriptome Analysis Revealed the Molecular Regulation Mechanism of Pigment and Reactive Oxygen Species Metabolism During the Stigma Development of *Carya cathayensis*

**DOI:** 10.3389/fpls.2022.881394

**Published:** 2022-05-09

**Authors:** Yulin Xing, Ketao Wang, Chunying Huang, Jianqin Huang, Yirui Zhao, Xiaolin Si, Yan Li

**Affiliations:** State Key Laboratory of Subtropical Silviculture, Zhejiang A&F University, Hangzhou, China

**Keywords:** *Carya cathayensis*, color change, gene expression, gene co-regulatory networks, pigment metabolism, ROS scavenging, stigma development, transcriptome analysis

## Abstract

Hickory (*Carya cathayensis* Sarg.) is a monoecious plant of the genus *Carya* of the Juglandaceae family. Its nuts contain a number of nutritional compounds and are deeply loved by consumers. Interestingly, it was observed that the color of hickory stigma changed obviously from blooming to mature. However, the molecular mechanism underlying color formation during stigma development and the biological significance of this phenomenon was mostly unknown. In this work, pigment content, reactive oxygen species (ROS) removal capacity, and transcriptome analysis of developing stigma of hickory at 4 differential sampling time points (S1, S2, S3, and S4) were performed to reveal the dynamic changes of related pigment, antioxidant capacity, and its internal molecular regulatory mechanism. It was found that total chlorophyll content was decreased slightly from S1 to S4, while total carotenoids content was increased from S1 to S3 but decreased gradually from S3 to S4. Total anthocyanin content continued to increase during the four periods of stigma development, reaching the highest level at the S4. Similarly, the antioxidant capacity of stigma was also gradually improved from S1 to S4. Furthermore, transcriptome analysis of developing hickory stigma identified 31,027 genes. Time-series analysis of gene expressions showed that these genes were divided into 12 clusters. Cluster 5 was enriched with some genes responsible for porphyrin and chlorophyll metabolism, carotenoid metabolism, and photosynthesis. Meanwhile, cluster 10 was enriched with genes related to flavonoid metabolism, including anthocyanin involved in ROS scavenging, and its related genes were mainly distributed in cluster 12. Based on the selected threshold values, a total of 10432 differentially expressed genes were screened out and enriched in the chlorophyll, carotenoid, anthocyanin, and ROS metabolism. The expression trends of these genes provided plausible explanations for the dynamic change of color and ROS level of hickory stigma with development. qRT-PCR analyses were basically consistent with the results of RNA-seq. The gene co-regulatory networks of pigment and ROS metabolism were further constructed and *MYB113* (CCA0887S0030) and *WRKY75* (CCA0573S0068) were predicted to be two core transcriptional regulators. These results provided in-depth evidence for revealing the molecular mechanism of color formation in hickory stigma and its biological significance.

## Introduction

The flower stigma is receptive portions of the female tissues that bind pollen and mediate tube migration into the style. It can be divided into dry and wet stigmas based on the structure. Angiosperms that produce trinucleate pollen usually have a dry stigma, while binucleate pollen usually interacts with a wet stigma. Although they have different morphological structures, all stigmas perform similar functions, including pollen capture and hydration, pollen tube guidance, and dispersal, all of which are crucial for successful fertilization and controlling seed yield (Nasrallah, 2000; Edlund et al., 2004).

Flower blossoms are products of sexual selection for traits that enhance mating success. Flowers vary in color, pattern, shape, and scent, which alone or in combination can act as signals for the attraction of animal pollinators (Pannell, 2017). Flower color plays an essential function in plant ecology and evolution by attracting animal pollinators (Grotewold, 2006), being a central feature of plant observation, and having a significant ornamental function (Xue et al., 2020). Due to the importance of color formation in angiosperm, especially in the ornamental plant, the biosynthetic pathways of pigment in color formation have been widely reported (Grotewold, 2006; Chen et al., 2016; Xia et al., 2021). Three chemically distinct groups of pigments, chlorophylls, carotenoids, and anthocyanins are widely distributed in plants and are the major pigments in flower color formation (Xue et al., 2020; Xia et al., 2021).

Chlorophyll is a critical component in almost all plants and makes plants green, which mainly participates in photosynthesis (Hörtensteiner, 2013). When photosynthetic organs are overexcited, such as under light stress conditions, chlorophyll can act as a photosensitizer, leading to cell damage and death to protect them (Apel and Hirt, 2004). After a lot of research, the pathway of chlorophyll metabolism has been very clear, which need the involvement of enzymes and transcription factors encoded by many genes, such as *glutamyl tRNA reductase (Glu-TR), glutamate-1-semialdehyde-2,1-amino mutase (GSA-AM), Porphobilinogen synthase (PBCS), bile chromogen dehydrogenase (PBGD), Uroporphyrinogen III synthase (UROS), Uroporphyrinogen III decarboxylase (UROD), Coproporphyrinogens III oxidase (CPOX), Protoporphyrinogen oxidase (PPOX), Magnesium chelatase H subunit (MgCh), Magnesium proto IX methyltransferase (MgPM), Mg-protoporphyrin IX monomethylester (MgPEC), 3,8-divinyl protochlorophyllide a 8-vinyl reductase (DVR), Protochlorophyllide oxidoreductase (POR), Chlorophyll synthase (CHLG), Chlorophyllide a oxygenase (CAO), hydroxy-Chl a reductasehydroxy-Chl a reductase (HCAR), non-yellow coloring1/NYC1-like (NYC1/NOL), metal-chelating substance (MCS), pheophytinase (PPH), pheophorbide a oxygenase (PAO), primary fluorescent Chl Catabolite (pFCC), and red Chl catabolite reductase (RCCR)* (Beale, 2005; Hörtensteiner, 2013).

Carotenoids are the second most abundant natural pigments on earth and are usually function in photosynthesis and photoprotection as well as growth and development (Tanaka and Ohmiya, 2008). Carotenoids are terpenoids chemicals and contribute to the formation of the yellow, orange, and red hue of most flowers (Tanaka and Ohmiya, 2008). It is the only precursor to vitamin A biosynthesis that is particularly beneficial in humans by promoting antioxidant activity and reducing age-related macular degeneration of the eye (Davies, 2007; Giuliano et al., 2008). Carotenoids can also quench O_2_ by a chemical mechanism involving their oxidation (Ramel et al., 2012). Its metabolism has been clarified, and some genes encoding enzymes and transcription factors are involved in the process. These genes included *1-deoxy-D-xylulose-5-phosphate synthase (DXS), 1-deoxy-D-xylulose-5-phosphate reductoisomerase (DXR), 2-c-methyl-d-erythritol 4-phosphate cytidyl transferase (MCT), 2-c-methyl-d-erythritol 4-phosphate cytidyl transferase (CMK), 2-C-Methyl-D-erythritol2,4-cyclodiphosphate synthase (MDS), 1-Hydroxy-2-methyl-2-butenyl 4-diphosphate synthase (HDS), 4-hydroxy-3-methylbut-2-enyl diphosphate reductase (HDR), isopentenyl diphosphate isomerase (IPI), geranylgeranyl diphosphate synthase (GGPPS)*, *phytoene synthase (PSY), phytoene desaturase (PDS)*, (*-carotene isomerase (ZISO))*, (*-carotene desaturase (ZDS)), lycopene-*(*-cyclase (LCY-E)), lycopene-*(*-cyclase (LCY-B)), LUTEIN DEFICIENT 5 (LUT5), LUTEIN DEFICIENT 1 (LUT1), violaxanthin de-epoxidase (ATVDE), zeaxanthin epoxidase (ZEP), carotene-*(*-hydroxylase (CHY-B)), carotene-*(*-hydroxylase (CHY-E)), violaxanthin de-epoxidase (VDE), Carotenoid Cleavage Dioxygenase (CCD), 9-cis-epoxycarotenoid dioxygenase (NCED),etc.* (Goodwin, 1971; Bramley, 1985; Vallabhaneni et al., 2010; Brandi et al., 2011; Fantini et al., 2013; Gao et al., 2021).

Flavonoids can produce the broadest spectrum of colors, ranging from pale yellow to blue-purple (Zhao and Tao, 2015). Anthocyanin is a type of flavonoid compound and is accumulated in different tissues of plants stimulated by drought, high light, and hormones (Zeng et al., 2010; Qi et al., 2011; Li et al., 2016). Anthocyanin is commonly found in various plants with widely biological functions, including protection against UV radiation, protecting leaf cells from photo-oxidative damage, and attracting pollinators (Winkel-Shirley, 2002; Bradshaw and Schemske, 2003; Falcone et al., 2012). Additionally, it can prevent cardiovascular disease, diabetes, aging, and cancer *via* scavenging ROS, bringing high health value to humans (He and Giusti, 2010; Li et al., 2022). After years of research by scientists, the synthetic pathway of flavonoids is also very clear, which involves *phenylalanine ammonia-lyase (PAL), cinnamate 4-hydroxylase (C4H), 4-coumaroyl-CoA ligase (4CL), catalysis of chalcone synthase (CHS), chalcone isomerase (CHI), flavanone-3-hydroxylase (F3H), flavanone-3*′*-hydroxylase (F3*′*H), flavanone-3*′*5*′*-hydroxylase (F3*′*5*′*H), anthocyanin synthase (ANS), Flavonol synthase (FLS)*, *dihydroflavonol reductase (DFR), flavonoid-3-o-glucosyltransferase (UFGT), etc.* (Chen et al., 2016). In *Arabidopsis*, a ternary protein complex (MYB-bHLH-WD40) is considered to regulate the expression of these anthocyanin biosynthetic genes (Zheng et al., 2019), and the R2R3-MYB transcription factors include *production of anthocyanin pigment 1 (PAP1, MYB75)*, *production of anthocyanin pigment 2 (PAP2, MYB90)*, *MYB113*, *MYB114*, *transparent testa 8 (TT8*), *enhancer of glabra (EGL3)*, and *transparent testa glabra 1 (TTG1)* (Walker et al., 1999; Nesi et al., 2000; Gonzalez et al., 2008; Jaakola, 2013).

Reactive oxygen species plays an essential role in diverse physiological processes. It exists in various forms in the aerobic environment, including superoxide, hydroxyl radicals, and other radical molecules (Mittler, 2017). In plants, ROS is produced mainly in chloroplasts, mitochondria, and peroxisomes continuously (Elstner, 1991; Rogers and Munné-Bosch, 2016). The ROS metabolism in plant cells is an intricate network involving many enzymatic and metabolite elements. It has dual roles in plants, promoting growth and development and playing a defensive role, but also producing toxic by-products of oxygen metabolism that lead to plant senescence (Mittler et al., 2004). In order to suppress the excessive accumulation of ROS, plants have evolved a variety of scavenging mechanisms, including a variety of ROS scavenging enzymes and non-enzymatic antioxidant compounds. For example, it was found that post-harvest UV-C exposure enhanced total anthocyanins and phenolic compounds in stored strawberries to scavenging the ROS, acting as a shield against UV-B (Raven, 2000; Severo et al., 2015; Xu Y. et al., 2017).

Hickory (*Carya cathayensis* Sarg.) is a deciduous tree of the genus *Carya* in the Juglandaceae family, endemic to China, mainly distributed in Tianmu mountainous area at the border of Zhejiang and Anhui provinces, integrating economic value and ecological value. Hickory is monoecious, with male flowers in catkins, inflorescence pendulous, and no tepal, and male flower buds usually begin to differentiate from mid to late May, then enter dormancy until late July (Huang et al., 2007, 2013). The female flowers show short spikes without tepal. Its buds begin to differentiate in early April, forming bracteoles and pistillate primordia, entering bud morphological differentiation in mid-April (Wang et al., 2012; Shen et al., 2014). Interesting, the hickory is a wind-pollinated plant, while the changing pattern of the color of stigma shows the characteristics of insect pollination, different from typical wind-pollinated plants. The color of pistil stigma varies markedly from opening to mature, in turn, presenting green, light red, bright red, purple-red, and, dark purple. During the pollination period, the stigma is bright red, bearing downy glands, and secretes mucus to facilitate the reception of pollen (Xia, 2006). Nevertheless, the reason for this color change in the hickory stigma is unclear. Whether this phenomenon is a protective mechanism of the plant itself against ROS accumulation or a feature of evolution is worth studying, which will be beneficial to solving the problem of the low yield of a hickory nut.

In this study, hickory stigma at different developmental periods offered ideal materials for revealing the molecular regulatory mechanism underlying the color changes of stigma and the biological significance of this phenomenon. By microplate assay, transcriptome sequence, and qRT-PCR, we were to: (1) measure the levels of chlorophylls, anthocyanins and, carotenoids of hickory stigma during the development; (2) determine the 1,1-Diphenyl-2-picrylhydrazyl radical 2,2-Diphenyl-1-(2,4,6-trinitrophenyl) hydrazyl (DPPH) and 2, 2’-azino-bis(3-ethylbenzothiazoline-6-sulfonic acid) (ABTS) radical scavenging capacity of stigma with development; (3) excavate the key differential genes with specific expression required for the color change and ROS metabolism during the hickory stigma development; (4) analyze the expression levels of genes required for pigment formation of stigma; (5) construct the gene co-regulatory networks between pigment and ROS metabolism. With these studies, the molecular mechanism of color formation and change in hickory stigma and its biological significance will be deeply understood, providing important theoretical guidance for the improvement of hickory nut yields in the new future.

## Materials and Methods

### Plant Material

The stigmas of hickory were collected from the experimental base in Linglong Mountain, Lin’an, China (119°38′51″E, 30°12′39″N, elevation: 119 m). A total of six hickory trees with similar growth and good vigor were selected, and female flowers were collected every 2–3 days from late April, according to the developmental state. Also, 4 sampling time points were selected, noted as S1, S2, S3, and S4, respectively. The pistillate flowers were taken back to the laboratory immediately after collection. After the sepals were removed, the stigma and ovary parts were separated, placed in liquid nitrogen for freezing, and then stored at −80°C in the refrigerator for pigment determination and RNA extraction for transcriptome sequencing. Each stage (S1 to S4) had three biological replicates.

### Total Chlorophyll and Carotenoid Content Measurement

The extraction and analysis of the total content of chlorophylls and carotenoids were determined according to the method described (Lichtenthaler, 1987; Xue et al., 2020). First, 20 mg of freeze-dried hickory stigma powder was added to 1,000 μl 95%(v/v) ethanol/water solution. After 24 h, it was centrifuged at 5,000 × *g* for 10 min, measured the absorbance at 652, 665, and 470 nm of the supernatants using an enzyme-labeled instrument (Tecan Spark^®^, Swiss). The following equations were used to calculate total chlorophyll and carotenoid contents:

Chl a (μg/g) = (16.29A_665_–8.54A_652_)*10;

Chl b (μg/g) = (30.66A_652_–13.58A_665_)*10;

Total Chl (μg/g) = Chl a + Chlb;

Total carotenoids (μg/g) = (1000A_470_–1.63Chl a–104.96Chl b)÷221*10

### Total Anthocyanin Content Measurement

The extraction and analysis of the total content of anthocyanin were performed using the pH-differential method (Wrolstad and Giusti, 2001; Xue et al., 2020). First, 4 stages of the stigmas were placed in a lyophilizer (Christ Alpha 2–4 LD plus, Germany) for 48 h, and then grind thoroughly to powder. Next, 25 milligrams of freeze-dried hickory stigma powder were added to 250 μL extraction buffer (Trifluoroacetic acid: formic acid: water: ethanol = 1:2:27:70, v/v), and the mixture was stood 24 h at 4°C in the dark. Following centrifugation at 12,000 rpm for 10 min, the supernatant was transferred to a fresh tube. Next, 100 μL supernatant was mixed with 900 μL.025 mol potassium chloride buffer (pH = 1.0), and another 100 μL supernatant was mixed with 900 μL.4 mol sodium acetate buffer (pH = 4.5). The mixture was stood for 1.5 h (complete sedimentation) at room temperature. The absorption of the mixture was recorded at 510 and 700 nm using the enzyme-labeled instrument with 3 repetitions. Finally, total anthocyanin content was calculated based on the molar absorbance of a cyanidin-3-glucoside standard by the equation:

total anthocyanins (μg/g) = (A × MW × DF × 10000)/(ε × L), where*A* = (*A*_510_−*A*_700_)_*pH*1.0_−(*A*_510_−*A*_700_)_*pH*4.5_, ε = 26900 L⋅mol^−1^cm^−1^(extinction coefficient of cyanidin-3-glucoside at 510 nm), L = path length, MW = molecular weight of cyanidin-3-glucoside, DF = dilution factor.

### Antioxidant Capacity Measurement

In this experiment, 300 ul anhydrous ethanol was added to a 30 mg sample, sonicated for 3 h, and then macerated overnight. The extract was centrifuged at 8000 rpm for 10 min, and the supernatant was prepared at a concentration of 100 mg/ml (Burits and Bucar, 2000).

The DPPH radical scavenging capacity of hickory female stigma was determined by referring to the method with minor modifications (Burits and Bucar, 2000). The extracts were diluted to 0.25, 0.5, 1, 1.5, and 2 mg/ml with anhydrous ethanol and prepared for use later. Took 500 ul of the diluted extract in a test tube, added 500 ul of.2 mM DPPH free radical ethanol solution, reacted at room temperature, avoided light for 30 min, and then measured the absorbance at 517 nm using an enzyme marker. The absorbance Ac of 500 ul of DPPH free radical ethanol solution mixed with 500 ul of ethanol was measured simultaneously. The IC50 (concentration of samples scavenging 50% of DPPH free radicals) was used to evaluate the magnitude of antioxidant activity of hickory female flower stigma extracts from four periods. The lower the IC50 value, the higher the antioxidant activity.

Clearance rate (%) = (A_*c*_ – A_*s*_)/A_*c*_ × 100%

The ABTS radical scavenging capacity of hickory female flower stigma was determined according to the method (Petretto et al., 2016). First, ABTS radical stock solution was prepared by mixing 7 mmol/L ABTS solution with 2.45 mmol/L potassium persulfate at a 1:1 ratio by volume and left overnight. The prepared ABTS radical solution was diluted with anhydrous ethanol to produce an absorbance value of 0.7 ± 0.02 at 734 nm. Then, 50 μL of the extract dilutions (0.25, 0.5, 1, 1.5, and 2 mg/mL) were mixed with 1 ml of ABTS radical solution, and the absorbance values were measured at 734 nm after a 30-min reaction at room temperature and protected from light.

Clearance rate (%) = (A_*c*_ – A_*s*_)/A_*c*_ × 100%

Ac is the absorbance value of anhydrous ethanol mixed with ABTS radical solution, and As is the absorbance value of the extract mixed with the solution with ABTS radical.

### Total RNA Extraction, mRNA Library Construction, and Sequencing

Samples were immediately frozen in liquid nitrogen and stored at –80°C for RNA-sequence analysis. Total RNA was extracted from female stigma at different developmental time points using CTAB-PBIOZOL reagent and ethanol precipitation. After extracting, the concentration, 28S/18S and RIN/RQN of total RNA were detected by Agilent 2100, and OD260/280 and OD260/230 were quantified by the NanoDrop. The construction of cDNA libraries and transcriptome sequencing were completed by BGI Technology (Shenzhen, China). First, total RNA was disposed of by mRNA enrichment using Oligo (dT)-attached magnetic beads. The mRNA was further broken using the fragment buffer after ligating sequence adapters. PCR amplification and cyclizing were used to get a single-strand cDNA library. The DNBSEQ platform performed the sequencing after the library was certified. All of the experiments were performed with three replicates. Raw reads were obtained from the sequencing, and the low-quality reads with adaptors were processed to obtain clean reads.

### RNA-Seq Data Analysis

Raw reads obtained were filtered with SOAPnuke (version 1.4) (Li et al., 2008) to filter out low-quality reads, contaminated junctions, and high levels of unknown base N. The clean reads were obtained and stored in FASTQ format, then aligned to the reference genome (hickory genome), using Hierarchical Indexing for Spliced Alignment of Transcripts (HISAT2 version 2.1) (Kim et al., 2015), followed by Bowtie2 (version 2.2.5) to align the clean reads to the gene set (Langmead and Salzberg, 2012). RSEM was used to calculate gene expression levels for individual samples (v1.2.8) (Li and Dewey, 2011). Once the new transcripts are obtained, we added the new transcripts with potential protein-coding to the reference gene sequence to form a complete reference sequence and then calculate gene expression levels. Differential gene expression analysis was performed by DESeq2 (Love et al., 2014) with a *Q*-value < 0.05. Finally, multiple samples are tested for differentially expressed genes as required, and in-depth clustering and functional enrichment analyses of differentially expressed genes (DEGs) are performed.

### Identification and Analysis of Differentially Expressed Genes

The assembled unigenes were functionally annotated using the Kyoto Encyclopedia of Genes and Genomes (KEGG)^1^ and Gene Ontology (GO) databases. The Blast2GO program provided GO annotation. WEGO then generated GO classification maps. The biological processes and unigenes annotations of the pathway were analyzed using KEGG. The KEGG comprehensive database resource was compared with our data for analysis. DEGs were identified using the NOISeq method (Tarazona et al., 2015) based on the gene expression level of each sample with a fold change of at least 2, *Q*-value < 0.01, then were analyzed using RNAseq (3 biological replicates per group), characterized by gene ontology enrichment analysis.

### Transcription Factor Prediction and Co-expression Network Construction

To characterize patterns across samples and identify interesting and highly covariant sets of genes, we constructed a gene co-expression network based on symbolic hybrid network types using the weighted correlation network analysis (WGCNA) package (Langfelder and Horvath, 2008; Linn et al., 2017). The WGCNA package was also used to construct gene co-expression networks from identified DEGs to establish the transcriptional regulatory structure of anthocyanin biosynthesis, carotenoid biosynthesis, chlorophyll metabolism, and ROS metabolism. The network was visualized using Cytoscape software (Smoot et al., 2011).

### QRT-PCR Analysis of Genes Involved in Selected Genes

Totals of 21, 18, and 21 unigenes related to chlorophyll, carotenoid, and anthocyanin metabolism were selected for qRT-PCR analysis. Total RNA was isolated from the stigma collected at four different developmental time points as previously described, and then transcribed 1000 ng of total RNA into cDNA for qRT-PCR using PrimeScript ™ RT reagent Kit with gDNA Eraser (removing genomic DNA) (Perfect Real Time) (TaKaRa, Japan) according to the instructions. The templet cDNA was diluted into 100 ng/μL (± 3 ng/μL). NCBI primer blast was used to design PCR primers to quantify gene expression involved in pigments formation, shown in Supplementary Table 1. The reaction system for RT-qPCR is as follow: ddH_2_O 3.8 μL, TB Green 5 μL, primerF.2 μL, primerR.2 μL, cDNA0.8 μL, with 3 repetitions. All qRT-PCR assays were performed using TB Green^®^ Premix Ex Taq™ (Tli RNaseH Plus) (TaKaRa, Japan) in a CFX96 Touch™ Real-Time PCR Detection System (BIO-RAD, United States) with the following reaction conditions: 95°C for the 30 s and 40 cycles of amplification (95°C for 5 s, 60°C for 30 s). The relative expression levels of target genes were calculated using the 2^–ΔΔCt^ method against the internal control (Livak and Schmittgen, 2001), and the *CcUBC9-5* gene was used as a control to normalize the relative expression levels of target genes. Experiments were performed with three independent biological replicates and three technical replicates.

### Analysis of Protein Physicochemical Properties and Prediction of Subcellular Localization

The protein physicochemical properties, including protein length, isoelectric points (PI), molecular weight (MW), and grandaverage of hydropathicity (GRAVE), were predicted using ProtParam^2^. The ProtScale^3^, SignalP3^4^, and Plant-mPLoc^5^ were used to predict protein hydrophobic/hydrophilic, signal peptides, and subcellular localization, respectively.

### Statistical Analysis

Statistical analysis was carried out with one-way ANOVA in SPSS17 software (ANCOVA; SPSS17, SPSS Inc., Chicago IL, United States). Significant differences among treatments were obtained according to *P*-values determined by Tukey’s honestly significant difference (Tukey’s HSD) with HSD (*P* < 0.05). All the experiments were repeated 3 times, and the results were presented as mean ± standard deviation (*SD*).

## Results

### Dynamic Changes in the Contents of Total Anthocyanin, Chlorophyll, and Carotenoid

**FIGURE 1:**
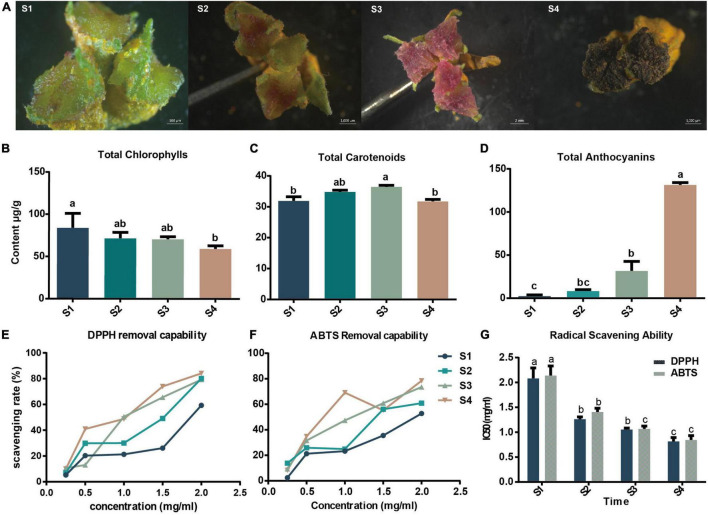
Pigmentation and phenotypes of hickory stigmas at the four flowering stages. **(A)** Morphological characteristics of hickory stigma during flower development (S1-S4). **(B–D)** Dynamic changes in the content of total chlorophylls, carotenoids, and total anthocyanins (cyanidin-3-glucoside used as the standard), expressed as μg/g dry weight (DW). **(E–F)** Differences in DPPH and ABTS radical scavenging ability in different concentrations of sample extracts expressed as mg/ml. **(G)** Differences in DPPH and ABTS scavenging capacity at the 4 different stages. Results are mean ± *SD* of three parallel measurements. Different letters correspond to significant differences (*P* < 0.05, Tukey’s HSD) by ANOVA.

During the development of the hickory flower, the stigma color changed obviously from green to dark-purple (S1: green, S2: light-purple red, S3: dark-purple red, S4: dark purple) (Figure 1A). To clarify the pigment components and their dynamic change, total chlorophyll, carotenoid, and anthocyanin at four stages of stigma development were further measured. It was shown that total chlorophyll content was relatively high overall and showed a continuous slight downtrend with stigma development (Figure 1B), while total carotenoid content was increased from S1 to S3 and reached the highest value of 36.4 (ug/g, while decreased gradually from S3 to S4 (Figure 1C). Total anthocyanin content gradually increased during the four periods of stigma development, sharped reaching the highest content (131.4 ug/g, with cyanidin-3-glucoside used as the standard) at the S4 (Figure 1D). These results suggest that the color change of the stigma of hickory flowers is due to the joint action of these pigments. The green color at the early stage is mainly caused by chlorophyll. In the middle and late stages, the accumulation of carotenoid and anthocyanin gradually makes the stigma appear red and purple.

### Differences in the Scavenging Capacity of Stigma for DPPH and ABTS Radicals

It is well-known that anthocyanins have a strong antioxidant capacity (Raven, 2000; Severo et al., 2015). The sharper changes of anthocyanins contents than the other two pigments from S3 to S4 inspire us to measure the ROS scavenging capacities. To explore the antioxidant capacity of stigma, removal-capacities of DPPH and ABTS radicals were further measured. It was found that the scavenging ability of DPPH radicals gradually increased with the process of stigma development and could reach 84.14% at the S4 period (Figure 1E). Similarly, the change of ABTS free radical scavenging capacity was close to that of DPPH, with 78.46% scavenging capacity at S4 (Figure 1F). It was also observed that DPPH and ABTS radical scavenging rates gradually increased with the increasing concentration of the hickory stigma extracts (Figures 1E,F). DPPH and ABTS IC50 could also be used to describe the oxidative scavenging capacity, the extracts from the stigma samples at S4 were more active in scavenging DPPH radicals, and their IC50s were significantly (*P* < 0.05) lower than those of the other three periods (S1, S2, and S3) (Figure 1G). The oxidative scavenging capacity obtained by the ABTS IC50 showed similar results. The highest clearance activity was observed during the S4 period and the lowest during the S1 period (Figure 1G).

### Overview of the Transcriptomic Analysis

**FIGURE 2:**
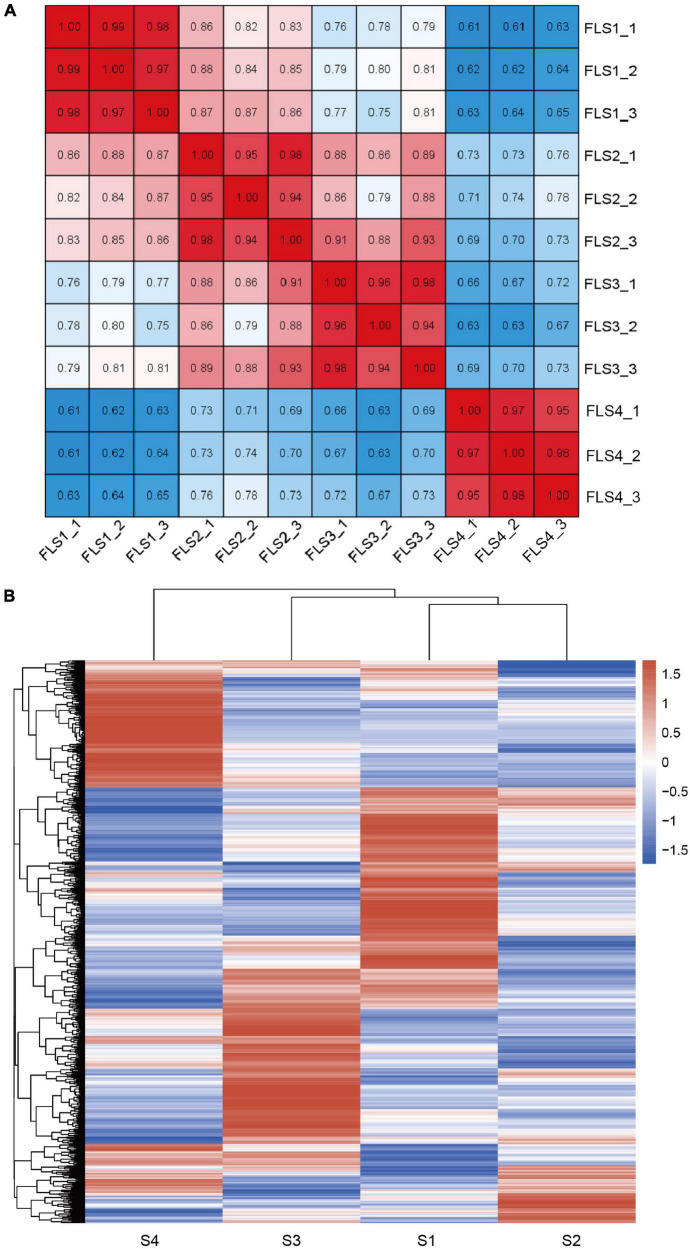
Basic survey of stigma transcriptome during flower development of hickory. **(A)** Pearson correlation results are based on all transcripts identified from RNA-seq. **(B)** Heatmaps of all genes by hierarchical cluster analysis (HCA) from RNA-seq.

**FIGURE 3:**
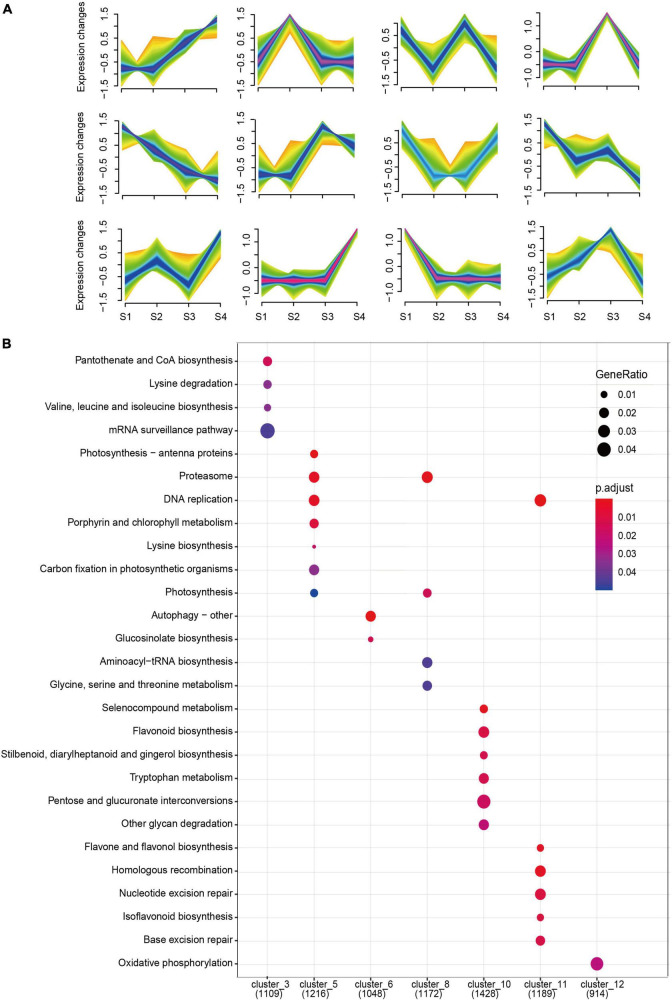
Analysis of 12 time-series clusters during stigma development of hickory. **(A)** Clustering analysis of gene expression patterns at different time points using fuzzy c-means algorithm (Mfuzz). **(B)** Kyoto Encyclopedia of Genes and Genomes (KEGG) analysis of 12 clusters.

To obtain a global transcriptome profile of developing stigma, twelve stigma samples at S1, S2, S3, and S4 were sequenced using the BGIseq500 platform. After removing adapter pollution, poly-N sequences, and low-quality reads, totals of 75.72 Gb clean bases were acquired, including an average of 45.24 Mb raw sequencing reads and 42.06 Mb clean reads, in each sample. The Q30 base percentage was greater than 90.78% and the average ratio of clean reads to raw reads was 92.97%. Using HISAT and Bowtie2, 93.43–94.53% of the clean reads could be mapped to the genome of hickory, 56.9–62.31% could be mapped to the genome of hickory uniquely. 67.88–71.17% (total), 52.83–54.87% (unique), and the clean reads were mapped to the hickory genes per sample, so that 31,027 genes were identified (Supplementary Table 2). Principal components analysis (PCA) showed that the sample clusters with high similarity converged, and the 4 sampling time points were separated without an outlier sample (Supplementary Figure 1). Based on the expression levels of all genes in every two samples, the Pearson correlation coefficient (PCC) was calculated and the value was at least 0.94, suggesting that the repeatability of the sample is very good (Figure 2A). Moreover, Hierarchical cluster analysis (HCA) was accompanied and displayed in the form of heatmaps plotted by a heatmap package in R software (Figure 2B), showing that significant differences in the expression levels of genes occurred between the sample groups. Using Mfuzz, twelve time-related gene clusters were obtained based on the similar expression patterns of some genes during stigma development (Figure 3A and Supplementary Table 3). KEGG analysis showed that some genes in cluster 5 were associated with “photosynthesis-antenna proteins,” “porphyrin and chlorophyll metabolism,” “carbon fixation in a photosynthetic organism,” “photosynthesis,” and “carotenoids metabolism,” with gradual downtrends from S1 to S4 (Figure 3B and Supplementary Table 4). These results indicated that photosynthesis mainly dominated in the pre-developmental period. Furthermore, the “Flavonoid biosynthesis” pathway was enriched in cluster 10, with a smooth trend in the pre-period, and increased significantly from S3 to S4 (Figure 3B and Supplementary Table 4). Other flavonoids, such as flavone, flavonol, and isoflavonoid, were found to be enriched in cluster 11, with a drastic reduction tendency from S1 to S2 but a slight change from S2 to S4 (Figure 3B and Supplementary Table 4). Notably, it was also found that oxidative phosphorylation-related genes were clustered in cluster 12, which showed a trend of increasing first from S1to S3 and then decreasing from S3 to S4 (Figure 3B and Supplementary Table 4).

**FIGURE 4:**
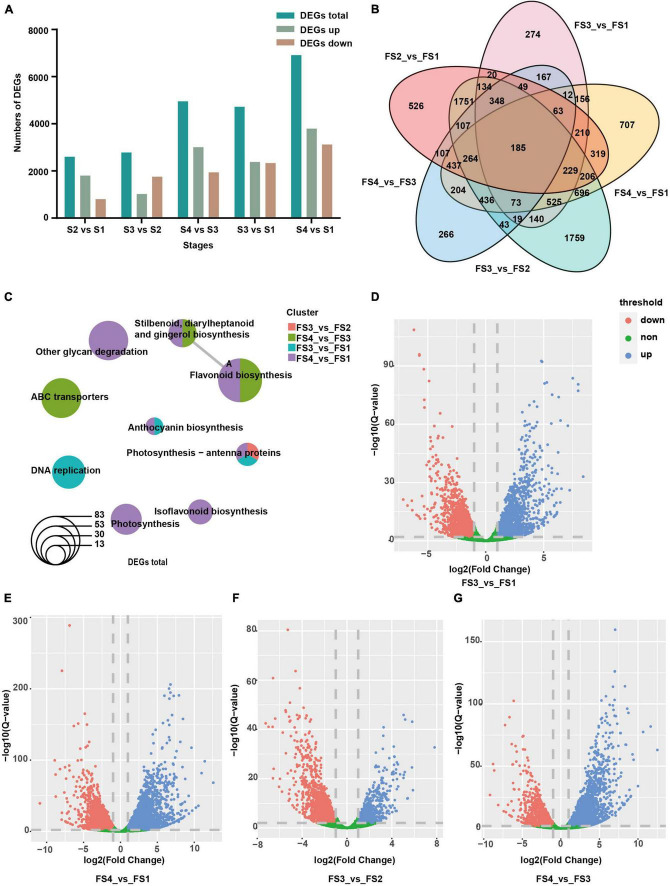
**(A)** Overview of DEGs and their pathway enrichment based on KEGG analysis. Numbers of differentially expressed genes (DEGs) in five comparisons; **(B)** Venn diagram of the number of DEGs revealed by five comparisons; **(C)** KEGG enrichment of all DEGs; **(D–G)** Volcanic maps for DEGs in four comparisons enriched by KEGG. Red dots, downregulated genes. Blue dots, upregulated genes. Green dots, non-differential genes.

Based on the selected threshold values of fold change with at least 2 (*Q*-value < 0.01, *P*-value of the cutoff standard < 0.05), a total of 10,432 DEGs were screened out in all the pair-wise comparison groups. In these DEGs, 2,604 (S2 vs. S1), 2,780 (S3 vs. S2) and 4,955 (S4 vs. S3), 4722(S3 vs. S1), and 6915 (S4 vs. S1) DEGs were counted out, including 1,801, 1026, 3011, 2383 and 3795 upregulated genes and 803, 1,754, 1,944, 2339 and 3120 downregulated genes, respectively (Figure 4A). In addition, Venn diagrams were mapped to figure out the relationship between the DEGs in the above comparison groups, indicating that a total of 185 DEGs existed in all five comparison groups (Figure 4B). These results suggested that there were dynamic changes in identified genes during stigma development and the active transcription occurred during the late period. KEGG analysis indicated that all identified DEGs were enriched in 15 pathways (Supplementary Table 5). It was also found that these DEGs enriched in the “Flavonoid biosynthesis” pathway (S4 vs. S1, S4 vs. S3, ko00941), “Photosynthesis-antenna proteins” (S3 vs. S1, S3 vs. S2, and S4 vs. S1, ko00196) pathways, “Photosynthesis” (S4 vs. S1, ko00195) pathway, “anthocyanin biosynthesis pathway” (S3 vs. S1, and S4 vs. S1, ko00942), “carotenoid biosynthesis pathways” (S4 vs. S1, ko00906) and ABC transporters (S4 vs. S3, ko02010) (Figure 4C and Supplementary Table 5). Furthermore, volcanic maps in S3 vs. S1, S3 vs. S2, S4 vs. S1, and S4 vs. S3 were drawn, intuitively showing the up-regulated genes (blue dots), down-regulated genes (red genes), and non-differential genes (green dots) (Figures 4D-G). These results indicated that the genetic manifestations of these pathways were active at the middle and late stages of female stigma development, offering a clear direction for elucidating the internal molecule mechanisms of the color formation and dynamic changes.

### Identification and Verification of Genes in Pigment Metabolism Pathways and Their Expression Analysis

**FIGURE 5:**
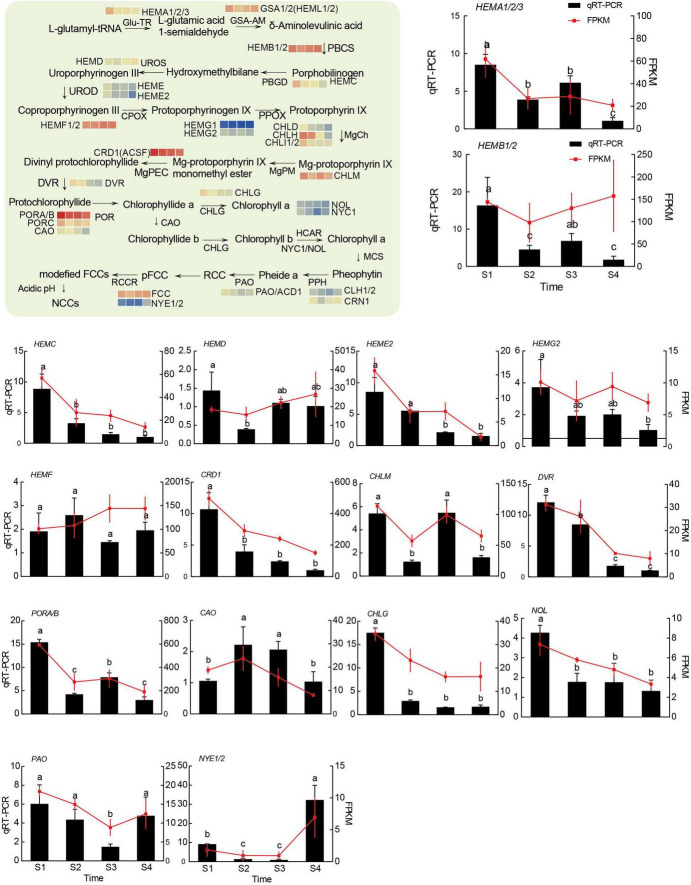
The model diagram of the main chlorophyll biosynthesis pathway, and transcriptional analysis of and qRT-PCR assays of 16 genes related to chlorophyll biosynthesis. R-package heatmaps were used to plot the expression heatmap of these genes. The heatmap data were homogenized by log2 (FPKM). The relative expression levels of target genes were calculated based on the 2^–ΔΔCt^ method against the internal control, and the *CcUBC9-5* gene was used as a control. Experiments were performed with three independent biological replicates and three technical replicates. Different letters showed significant differences (*P* < 0.05, Tukey’s HSD) by ANOVA among groups.

**FIGURE 6:**
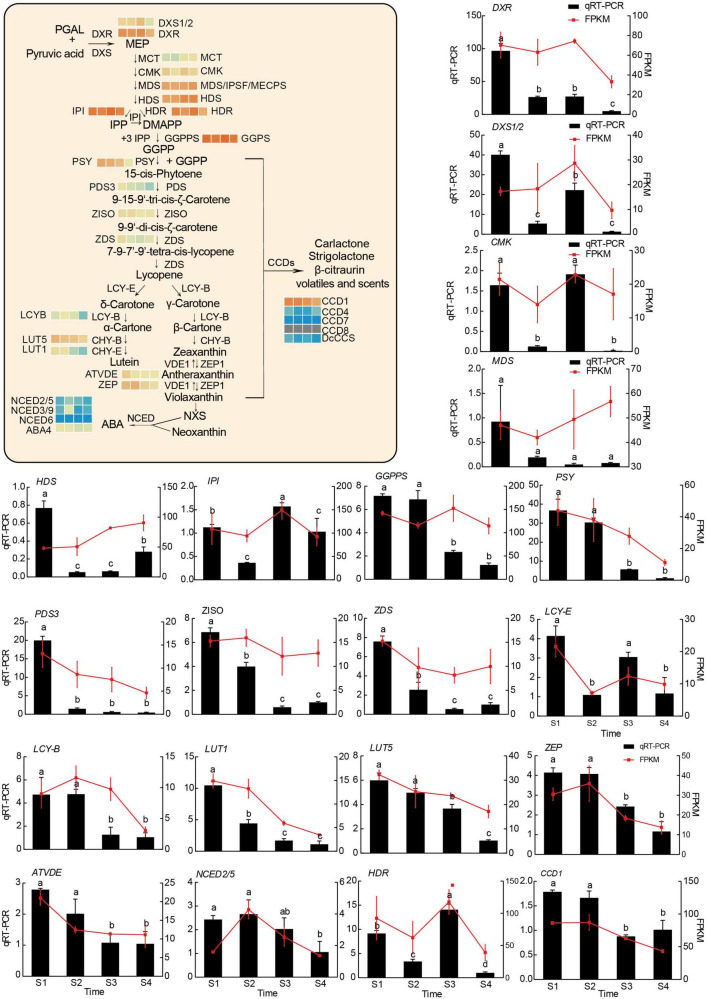
The model diagram of the main carotenoid metabolism pathway, and transcriptional analysis of and qRT-PCR assays of 20 genes associated with carotenoid metabolism. R-package heatmaps were applied to map the expression heatmap of these genes. The heatmap data were homogenized by log2 (FPKM). The relative expression levels of target genes were calculated by the 2^–ΔΔCt^ method against the internal control (*CcUBC9-5*). Experiments were conducted with three independent biological replicates and three technical replicates. Different letters indicated significant differences (*P* < 0.05, Tukey’s HSD) by ANOVA among groups.

**FIGURE 7:**
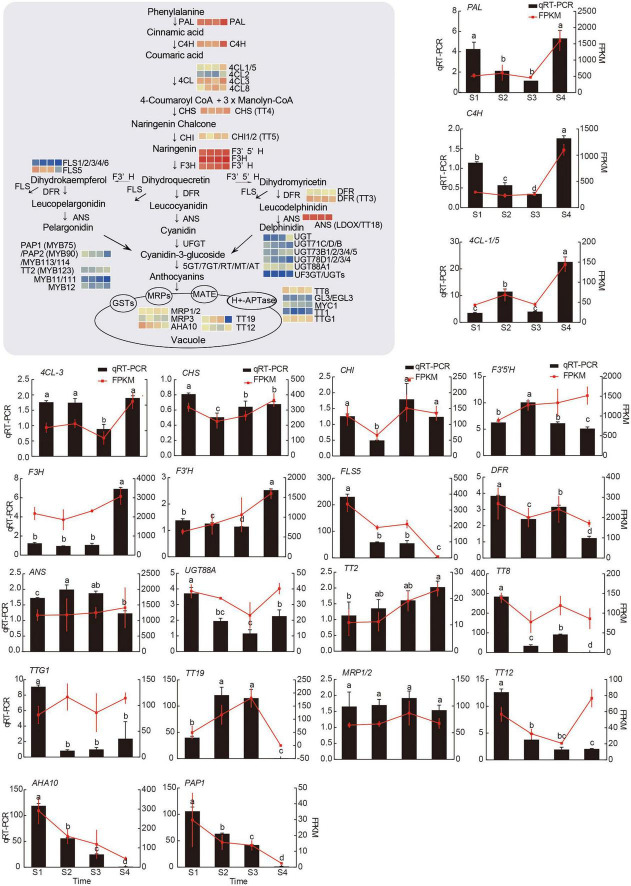
The model diagram of the main anthocyanin biosynthesis pathway, and transcriptional analysis of and qRT-PCR assays of 21 genes associated with anthocyanins metabolism. R-package heatmaps were applied to map the expression heatmap of these genes. The heatmap data were homogenized by log2 (FPKM). The relative expression levels of target genes were calculated using the 2^–ΔΔCt^ method and the *CcUBC9-5* was taken as an internal control. Experiments were carried out with three independent biological replicates and three technical replicates. Different letters represented significant differences (*P* < 0.05, Tukey’s HSD) by ANOVA among groups.

The above results indicated that chlorophyll, carotenoid, and flavonoid, including anthocyanins, may be involved in color formation. According to the whole genomic information of the hickory and KEGG database, the metabolism pathways of chlorophyll, carotenoid, and flavonoid, including anthocyanins, were drawn and genes encoding enzymes and transcription factors were annotated in these pathways (Figures 5–7 and Supplementary Table 6). Flower color-related genes with critical roles were further analyzed and 91 genes were screened out. Among these genes, 27 genes are required for chlorophyll metabolism, 28 genes were related to carotenoid metabolism and 36 genes were associated with anthocyanins metabolism. Among these genes, 21, 19, 22, and 18 from S1, S2, S3, and S4, respectively, had FPKM values of over 100. In the chlorophyll metabolic pathways, some genes, such as *GSA, HEMB, HEMD, HEMF1/2, CLH1/2, FCC*, and *NYE1*, were upregulated, while *HEMA, HEMC, HEME, HEMG, CHLD, CHLH, CHLI, CRD, DVR, POR, CAO, CHLG, NYC1*, and *PAO*, were downregulated (Figure 5 and Supplementary Table 6). In the carotenoid biosynthesis pathway, some genes, including *DXS, MCT, CMK, MDS, HDS, GGPPS*, and the downregulated genes *DXR, PSY, PDS, ZISO, ZDS, LCY-E, LCY-B, LUT5, LUT1, ATVDE*, and *ZEP*, were shown to be upregulated (Figure 6 and Supplementary Table 6).

Based on the results showing that there were greater changes in anthocyanin level and strong antioxidant capacity, a detailed analysis of the expression patterns of anthocyanin-related genes was conducted according to the data in Supplementary Table 6. Through the analysis of the synthesis pathway, we found that the expression patterns of most genes, including *PAL, C4H, 4CL1/5, 4CL2, 4CL3, CHS, F3H, F3′H, F3′5 ′H, DFR, ANS*, *UF3GTs, UGT, UGT73B1/B2/B3/B4/5*, and *UGT88A1*, were basically consistent, showing upward trends from S3 to S4, contributing to rapid accumulation of anthocyanins at a late stage of stigma development. It was also observed that the *FLS* gene required for quercetin-Der, Kaempferol-Der, and myricetin-Der biosynthesis showed the opposite trends as the above-described genes, suggesting that more premise substances enter into the leucocyanidin, leucopelargonidin, and leucodelphin for anthocyanins accumulation. After the anthocyanins biosynthesis is finished, they will be transported into the vacuole, involving some transporter proteins. It could be seen that the expression trends of *TT2* coding transporter proteins were similar to that of anthocyanins biosynthesis genes, while others, including *TT19, MRPs*, and *AHA10*, showed the opposite trends from S3 to S4. This is an indication that *TT2* may be the main transporter for anthocyanins in hickory stigma. In addition, previous studies have reported that the anthocyanin metabolism pathway was regulated by a suite of transcription factors that include MYB, bHLH, and WD-repeat proteins (Walker et al., 1999; Nesi et al., 2000; Gonzalez et al., 2008; Jaakola, 2013). It was observed that some TFs, including *TT2*, *TT1*, and *TTG1*, showed upward trends from S3 to S4, while others like *MYB75*, *MYB11*, *MYB12*, *MYC1*, *TT8*, and *GL3* were on the contrary. This may be attributed to the fact that these TFs usually form protein complexes to function. To regulate the expression of anthocyanin-related genes, these TFs need to be transcribed earlier. When the data were homogenized *via* Z-score normalization, the trends in gene expression became clearer (Supplementary Figure 3). Together, the expression trends of these pigment-related genes provided plausible explanations for the dynamic change of hickory stigma color with development.

To validate the results of RNA-seq, 20, 16, and 21 unigenes related to chlorophyll metabolism, carotenoid biosynthesis, and anthocyanin biosynthesis were selected for qRT-PCR analysis. As was shown in Figures 5–7, the results of the qRT-PCR were similar to the RNA-seq analysis, showing a high correlation, except for minor differences in some genes. These results further confirmed the reliability of the RNA-seq data in this study.

### Establishment and Analysis of Weighted Correlation Network Analysis Modules

**FIGURE 8:**
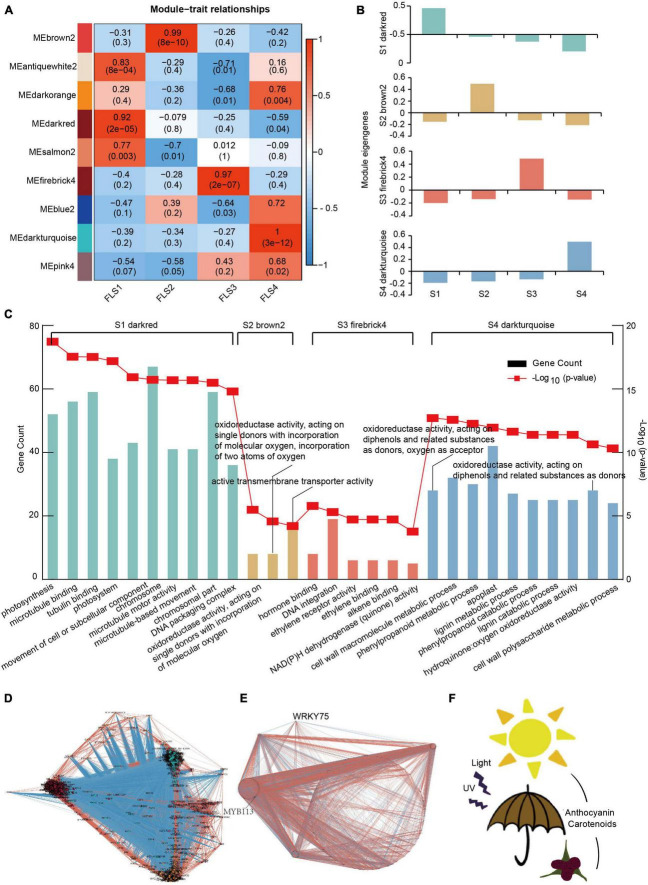
Establishment of WGCNA modules of the differentially expressed genes (DEGs) at the four stages of hickory stigma. **(A)** Module-trait correlations and DEGs network analysis established by WGCNA. Colors indicated the strength and direction of the correlation according to the color legend at the right, and the color scale represented the module-trait correlations from −1 to 1. The numbers in parentheses are the partial Pearson correlation coefficients and the corresponding *P*-values. **(B)** Module eigengenes of four hub modules. **(C)** GO analysis of four hub modules (darkred, brown2, firebrick4, and dark turquoise). **(D)** co-expression networks of anthocyanin, chlorophyll, carotenoid, ROS, and transcription factors in four modules. **(E)** co-expression networks of *MYB113 and WRKY75* and pigments, ROS, TFs with the FPKM more than 10 at any stage. **(F)** The working module of hickory stigmas is photo protected by forming pigments, such as anthocyanins, to scavenge reactive oxygen species.

To gain insight into the patterns of genetic association between different samples and further study the high covariate gene sets associated with pigment and ROS metabolism, we used WGCNA to construct a co-expressed gene network based on 10,432 DEGs (Supplementary Table 7). After removing the low expression genes, nine different gene modules were obtained by HCA and were demonstrated in different colors. Among nine modules, four significant modules were found through analyzing the relationship between modules and traits (Figure 8A). The darkred-, brown2-, firebrick4- and dark turquoise-modules were significantly correlated with the trait at S1 (PCC = 0.92, P = 2*e^–5^), S2 (PCC = 0.99, P = 8*e^–10^), S3 (PCC = 0.97, P = 2*e^–7^), and S4 (PCC = 1, P = 3*e^–12^), respectively. Figure 8B further showed the module eigengene in the four modules, which indicates these four modules were strongly interrelated in the four stages (Figure 8B). This result illustrated that the DEGs in the darkred, brown2, firebrick4, and the dark turquoise module were more important to correlate with the 4 stages during hickory stigma development. In the 4 hub modules, 17 chlorophyll-related DEGs and 12 carotenoid-related DEGs were mostly contained in the darkred module, while most anthocyanin biosynthesis-related DEGs were distributed in the dark turquoise module, with 112 DEGs (Supplementary Table 8). GO analysis further showed the “photosynthesis” and “photosystem” were enriched in the darkred module, which was closely related to S1 (Figure 8C and Supplementary Table 9). Notably, “oxidoreductase activity, acting on single donors with incorporation of molecular oxygen, GO:0016701,” “oxidoreductase activity, acting on single donors with incorporation of molecular oxygen, incorporation of two atoms of oxygen, GO:0016702,” and “active transmembrane transporter activity, GO:0022804” related pathway were concentrated on the brown2 module (Figure 8C and Supplementary Table 9). These related DEGs in the firebrick4 module were associated with “hormone-binding, GO:0042562,” “ethylene receptor activity, GO: 0038199,” “ethylene binding, GO:0051740” and “NAD(P)H dehydrogenase (quinone) activity, GO:0003955” (Figure 8C and Supplementary Table 9). In the dark turquoise module, these DEGs were involved in “oxidoreductase activity, acting on diphenols and related substances as donors, oxygen as acceptor, GO:0016682,” “phenylpropanoid metabolic process, GO:0009698” and hydroquinone: oxygen oxidoreductase, GO:0052716” (Figure 8C and Supplementary Table 9). These results further suggested that pigment and ROS metabolism were very active during hickory stigma development.

### Co-expression Network of Genes Related to Reactive Oxygen Species and Pigments Metabolism

According to pigment and ROS metabolism as well as related transcription factors in 4 key modules, a co-expression network was further constructed (Figure 8D). It was found that this network included 23 chlorophyll-related genes, 19 carotenoid-related genes, 141 anthocyanin-related genes, 46 ROS-related genes, and 623 transcription factors (TFs) (Supplementary Table 10). These transcription factors belonged to different families, including MYB (99), AP2(52), bHLH (52), NAC(30), WRKY(28), C_2_H_2_(21), HB-HD-ZIP(20), bZIP9(19), Trihelix(18), and others (284) (Supplementary Figure 2). Notably, 7 transcription factors (6 in the MYB family and 1 in the bHLH family) were shown to be related to anthocyanin metabolism. Moreover, the number of connections between genes was also counted, ranging from 49 to 447 (Figure 8E and Supplementary Table 10). In the chlorophyll metabolism pathway, *HEME2* (CCA1503S0015) and *CAO* (CCA1377S0020) had relatively high connections with more than 400 connections. It was also found that the connections of only the *PSY* (CCA0887S0096) gene in carotenoid biosynthesis-related genes were over 400. In the anthocyanins metabolism pathway, genes with more than 400 connections were *FLS1* (CCA0006S0007), *4CL8* (CCA0881S0026), *F3′H* (CCA0743S0031), *F3′H* (CCA0507S0050), and *TT12* (TRANSPARENT TESTA 12-like, CCA1606S0005). In ROS metabolism, *GSTU18* (glutathione S-transferase TAU 18, CCA0747S0058) was shown to have over 400 connections. Among genes encoding all the transcription factors, there were 21 genes with more than 400 connections, of which, the gene expression difference of *WRKY75* (WRKY transcription factor 75, CCA0573S0068) was the largest during stigma development. Remarkably, *MYB113* (MYB transcription factor 113, CCA0887S0030) was more connected to other genes in 7 anthocyanin-related transcription factors. Based on this, we further mapped the co-expression network of these two transcription factors and genes related to pigment and ROS metabolism, which more clearly showed the regulatory relationship of transcription factors on them (Figure 8E). Using ProtParam, the physicochemical properties of the two transcription factors were further analyzed, as shown in Table 1. It was demonstrated that the protein length, pI, MW, and GRAVY of *WRKY75* and *MYB113* were 152 and 264 aa, 9.64 and 9.1, 17.64 and 30.21 kD, and –1.156 and –0.536, respectively. Both proteins were hydrophilic with no signal peptides. Furthermore, the subcellular localization prediction showed that they were both located in the nucleus. Taken together, these results provide in-depth guidance for revealing the transcriptional regulation of pigment and ROS metabolism in the future.

**TABLE 1:** Physicochemical properties of proteins of *WRKY75* and *MYB113* in hickory.

Gene	Gene ID	Length	PI	MW (kDa)	GRAVE	localization	Hydrophobic/hydrophilic	Signal peptides
*WRKY75*	CCA0573S0068	152	9.64	17.64	–1.156	nucleus	Hydrophilic	No
*MYB113*	CCA0887S0030	264	9.1	30.21	–0.536	nucleus	Hydrophilic	No

## Discussion

Darwin had noted that when the wind fertilizes a flower, it never has a gaily colored corolla. It means that the relationship between floral traits and pollinator behavior has been an important factor influencing the co-evolution of plants and pollinators (Chittka et al., 2001). Hickory is a monoecious plant of the genus *Carya* in the Juglandaceae, and its nuts are one of the most popular nuts among consumers because of their high nutritional value. It is a wind-pollinated plant, while the stigma of female flowers shows different colors with development. From opening to mature, the stigma color presents successively green, light-purple red, dark-purple red, dark purple (Figure 1A). Chlorophylls, carotenoids, and anthocyanins are three chemically diverse groups of pigments and the core members in flower color formation (Xue et al., 2020; Xia et al., 2021). Our present work showed that the stigma appeared green at the first stage (S1) when the chlorophyll content was higher than the other two pigments, gradually turning red and purple as the anthocyanin and carotenoid content increased (S2 to S3), and eventually getting dark purple due to the reduction of carotenoid content and sharp increase of anthocyanin content of from S3 to S4 (Figures 1A–D). These results suggested that chlorophyll, carotenoid, and anthocyanin were involved in the color formation of hickory stigma, supporting an identified possibility to consider that flavonoids and especially anthocyanins play many essential functions in reproductive tissues, but do not attract pollinators (Gould, 2004; Taylor and Grotewold, 2005). It was also shown that there were sharper changes in anthocyanin than that in other pigments from S3 to S4 (Figures 1B–D). Anthocyanins are known to have a strong ROS-removal capacity (Raven, 2000; Severo et al., 2015), stimulating our curiosity about the ROS elimination in hickory stigmas. Notably, the results of the oxidative scavenging ability of the sample extracts were consistent with the pigment change trends, especially the anthocyanin (Figures 1E–G). Therefore, it was speculated that this phenomenon was a protective mechanism of the plant itself against ROS accumulation.

In hickory, the whole genome has been sequenced and assembled, and the transcriptomes of several tissues have been sequenced (Huang et al., 2019). However, most studies have focused on male and female flower organ development and fruit formation stages (Huang et al., 2022), but there is no transcriptomic analysis of color changes in different flowering stages. In this research, RNA-seq technology was performed to analyze 4 sample stages with three replicates during hickory stigma development to investigate the model and mechanism of changes in transcriptional patterns associated with flower color.

Through time-series expression analysis, 12 clusters were observed (Figure 3A). KEGG analysis demonstrated cluster 5 was associated with porphyrin and chlorophyll metabolism, and the expression patterns were basically consistent with chlorophyll content and flower color (Figures 1B, 3B). In cluster 10, the “flavonoid biosynthesis” pathway was enriched, and the expression trends were similar to that of anthocyanin content, forming red and purple stigmas. In cluster 11, other flavonoids, such as flavone, flavonol, and isoflavonoid, were enriched with a drastic reduction tendency from S1 to S2 but slight changes from S2 to S4, which may be beneficial for more prerequisite substances of phenylpropane synthesis pathway to enter anthocyanin synthesis (Figures 1D, 3B). In cluster 12, oxidative phosphorylation-related genes were clustered and showed a trend of increasing from S1 to S3, which was similar to that of oxidative scavenging ability, proving some molecular evidence for ROS metabolism (Figures 1E–G, 3B). According to all genes, 10432 DEGs were found, including 6549 upregulated genes and 6181 downregulated genes (Figure 4A). KEGG analysis of these DEGs showed that 14 pathways were enriched, including “chlorophyll metabolism,” “carotenoid biosynthesis,” and “anthocyanin biosynthesis.” These results suggested that these genes required for the color formation were very active, offering some explanation for the dynamic changes of hickory stigma color.

The chlorophyll metabolism pathway had been well elucidated (Hörtensteiner, 2013). The chlorophyll metabolism pathway had been painted based on the whole genomic information of the hickory and KEGG database, and 27 DEGs were identified in this study (Figure 5 and Supplementary Table 6). According to RNA-seq analysis and qRT-PCR assays, most genes showed high expression at the beginning of development and decreased at later stages. In contrast, chlorophyll degradation genes showed higher expression at later stages. Similarly, this phenomenon has been verified in *Lonicera japonica* Thunb. and *Lilium* (Xu L. et al., 2017; Xue et al., 2020).

Carotenoids can make plants yellow, orange, and red (Nisar et al., 2015). In this study, 28 DEGs were identified, including the critical candidate enzymes and TFs related to carotenoid metabolism (Supplementary Table 6). The present study showed that most of the DEGs had higher expression levels at the middle stage (Figure 6), consistent with the determination of carotenoid content. Interestingly, based on WGCNA and co-expression network results, 4 of these genes were connected with ABA, one of the carotenoid degradation products (Figure 8A and Supplementary Table 10) (North et al., 2007). Therefore, it meant that the carotenoids participated in pigment formation in hickory stigma.

Based on the analysis of the three pigment content measurements, the anthocyanin content appeared to be a significantly sharper change (Figure 1D), so we believe that anthocyanin is not only a role pigment in the accumulation and color change of hickory stigma but also one of the reasons that may lead to other biological phenomena during the development process, which is worthy of attention. The anthocyanin synthesis pathway has been thoroughly investigated as a type of flavonoid compound (Ferrer et al., 2008). Using the KEGG analysis and selected criteria, the study had identified 36 DEGs associated with the anthocyanins pathway (Supplementary Table 6). According to the RNA-seq results, KEGG database, and previous studies, the anthocyanin synthesis, and transport pathway had been diagrammed, with the heatmaps of several role genes being plotted beside it (Figure 7), qRT-PCR assays had been used to qualify the role genes, and the trends of expression of the most consisted with transcriptome results (Figure 7). It has been reported that the accumulation of UV-absorbing pigments, especially flavonoids in epidermal tissues, was the main mechanism of photo-protection in plants against UV light (Agati and Tattini, 2010; Agati et al., 2013; Xu Y. et al., 2019). It has also been shown that the quercetin derivatives biosynthesis genes *CHS, CHI, F3H, FLS*, and *F3H* were present in lower terrestrial plants (Markham, 1988; Rausher, 2006), which were the most sensitive genes in the face of oxidative damage in modern terrestrial plants (Lin et al., 2003; Pollastri and Tattini, 2011). Notably, these genes have also been identified in anthocyanin-related DEGs. In May, the hickory flower begins to form, when strong sunlight hits the stigma surface, the young stigma can be directly and easily burnt. Therefore, it is likely that anthocyanin-related genes were activated by strong sunlight to promote the anthocyanin accumulation to counteract high-intensity light-induced oxidative damage by scavenging ROS.

Previous studies have been reported that some transcription factors (TFs) regulate the anthocyanin metabolism, some of which including MYB, bHLH, and WD-40 can be united to form complex functions (Gonzalez et al., 2008; Liu et al., 2016; Wang et al., 2019). So it is very important to find core TFs regulating anthocyanin and ROS metabolism. Based on WGCNA and co-expression network analysis (Figure 8A and Supplementary Table 10), 7 transcription factors related to anthocyanin synthesis, especially, *MYB113* (*CCA0887S0030*) had the most connectivity, suggesting that it was a core TF in this pathway, which have also been reported as a core regulator in anthocyanin synthesis in *Arabidopsis, Solanum tuberosum*, and *Solanum melongena* (Walker et al., 1999; Liu et al., 2016; Zhou et al., 2020).

Our results have also shown that the DEGs were clustered into nine different color modules by WGCNA, among which, four key modules (darkred, brown2, firebrick4, and dark turquoise) (Figure 8A) were found. Moreover, after investigating WGCNA, together with GO analysis (Figure 8C), the darkred module could cluster some genes responsible for “photosynthesis” and “photosystem,” and these genes were highly expressed at the early stage of female flower stigma development. This is an indication that the early stage stigma mainly performs photosynthesis to provide plant growth and development nutrients. Previous studies have reported that adjustment of growth and development to light conditions is usually established by changes in hormone levels (de Wit et al., 2016). As a classic plant hormone, Ethylene can regulate anthocyanin biosynthesis and ROS scavenging in a variety of plants (Ni et al., 2021). In *Arabidopsis*, *ERF4* and *ERF8* promote the accumulation of light-induced anthocyanins (Koyama and Sato, 2018). It has also been reported that ethylene can also regulate anthocyanin synthesis in ripe apple fruit through antagonism of the R2R3-MYB repressor *MYB17* and activators *MYB1* and *MdEIL1* (Wang et al., 2022). In the middle stage of hickory stigma development, the firebrick4 module was enriched in “hormone-binding,” “ethylene receptor activity,” and “ethylene binding” pathway, and related DEGs including some TFs have been sorted out, providing a new direction for our future study. At the late stages of flower stigma development, these DEGs were mainly enriched in “oxidoreductase activity,” “active transmembrane transporter activity,” “hormone-related,” and “phenylpropanoid metabolic process,” which was contributed to the balance of pigment and ROS accumulation. These results provide some molecular evidence for the scavenging rate of reactive oxygen radicals in the extracts of samples from different periods. So, it is likely that the stigma color change act on the ability to scavenge ROS to protect stigma against various abiotic and biotic stresses. Notably, the co-expression network also showed that *WRKY75* (CCA0573S0068) may be a core transcription factor involved in pigment and ROS metabolism. In *Arabidopsis*, *WRKY75* has been shown to induce H_2_O_2_ accumulation as a key way to accelerate the leaf senescence process (Li et al., 2012; Guo et al., 2017). In poplar, *WRKY75* was reported to reduce the reactive oxygen scavenging capacity of leaves under stress and negatively regulate salt and osmotic tolerance by modulating multiple physiological processes (Zhao et al., 2019). Previous studies have suggested that the WRKY structural domain can bind to the cis-acting element W-box (Devaiah et al., 2007). Further work will be done to verify the direct regulating relationship of *WRKY75* on the targeted gene with more W-box required for pigment and ROS metabolism, including *ATAF1, GSTL3*, and *GRF7*, using some molecular techniques.

## Conclusion

In conclusion, it was demonstrated that color changes during hickory female stigma development were due to the dynamic changes in the content of chlorophylls, carotenoids, and anthocyanins. The antioxidant capacities had similar changing trends with carotenoids from S1 to S3 and anthocyanins at all four stages during stigma development. Transcription analysis of developing stigma provided the comprehensive molecular mechanism for the dynamic changes in pigment content and antioxidant capacities. *MYB113* (CCA0887S0030) and *WRKY75* (CCA0573S0068) were further predicted to be two core transcriptional regulators responsible for pigment and ROS metabolism. These results suggested that one of the important biological significance of the color change of female flower stigma was the photo-protection and anti-oxidation, the color variation makes the appearance of this ability in plant stigmas a possibility, and corresponding models have also been proposed to explain this possibility as well (Figure 8F). At present, we have not yet found any link between stigma color and the pollination process in hickory, and some additional analyses are still needed to verify this possibility in the new future.

## Data Availability Statement

The datasets presented in this study can be found in online repositories. The names of the repository/repositories and accession number(s) can be found below: National Center for Biotechnology Information (NCBI) BioProject database under accession number PRJNA810757.

## Author Contributions

YL, YX, and JH conceived and designed the study. KW and YX analyzed the data. YX performed the experiments. YL and YX wrote the manuscript. YL, JH, and KW edited and reviewed the writing. All authors have read and approved this manuscript.

## Conflict of Interest

The authors declare that the research was conducted in the absence of any commercial or financial relationships that could be construed as a potential conflict of interest.

## Publisher’s Note

All claims expressed in this article are solely those of the authors and do not necessarily represent those of their affiliated organizations, or those of the publisher, the editors and the reviewers. Any product that may be evaluated in this article, or claim that may be made by its manufacturer, is not guaranteed or endorsed by the publisher.

## Footnotes

1
https://www.kegg.jp/

2
https://web.expasy.org/protparam/

3
https://web.expasy.org/protscale/

4
https://services.healthtech.dtu.dk/service.php?SignalP-3.0

5
http://www.csbio.sjtu.edu.cn/bioinf/plant-multi/

## Glossary

*Glu-TR, glutamyl tRNA reductase; GSA-AM, glutamate-1-semialdehyde-2,1-amino mutase; PBCS, Porphobilinogen synthase; PBGD, bile chromogen dehydrogenase; UROS, Uroporphyrinogen III synthase; UROD, Uroporphyrinogen III decarboxylase; CPOX, Coproporhyrinogen III oxidase; PPOX, Protoporphyrinogen oxidase; MgCh, Magnesium chelatase H subunit; MgPM, Magnesium proto *IX* methyltransferase; MgPEC, Mg-protoporphyrin *IX* monomethylester; DVR, 3,8-divinyl protochlorophyllide a 8-vinyl reductase; POR, Protochlorophyllide oxidoreductase; CHLG, Chlorophyll synthase; CAO, Chlorophyllide a oxygenase; HCAR, hydroxy-Chl a reductasehydroxy-Chl a reductase; NYC1/NOL, non-yellow coloring1/NYC1-like; MCS, metal chelating substance; PPH, pheophytinase; PAO, pheophorbide a oxygenase; pFCC, primary fluorescent Chl Catabolite; RCCR, red Chl catabolite reductase; DXS, 1-deoxy-D-xylulose-5-phosphate synthase; DXR, 1-deoxy-D-xylulose-5-phosphate reductoisomerase; MCT, 2-c-methyl-d-erythritol 4-phosphate cytidyl transferase; CMK, 2-c-methyl-d-erythritol 4-phosphate cytidyl transferase; MDS, 2-C-Methyl-D-erythritol2,4-cyclodiphosphate synthase; HDS, 1-Hydroxy-2-methyl-2-butenyl 4-diphosphate synthase; HDR, 4-hydroxy-3-methylbut-2-enyl diphosphate reductase; IPI, isopentenyl diphosphate isomerase; GGPPS, geranylgeranyl diphosphate synthase; PSY, phytoene synthase; PDS, phytoene desaturase; ZISO, -carotene isomerase; ZDS, -carotene desaturase; LCY-E, lycopene-(-cyclase); LCY-B, lycopene-(-cyclase); LUT5, LUTEIN DEFICIENT 5; LUT1, LUTEIN DEFICIENT 1; ATVDE, violaxanthin de-epoxidase; ZEP, zeaxanthin epoxidase; CHY-B, carotene-(-hydroxylase); CHY-E, carotene-(-hydroxylase); VDE, violaxanthin de-epoxidase; CCD, Carotenoid Cleavage Dioxygenase; NCED, 9-cis-epoxycarotenoid dioxygenase; PAL, phenylalanine ammonia lyase; C4H, cinnamate 4-hydroxylase; 4CL, 4-coumaroyl-CoA ligase; CHS(catalysis of chalcone synthase; CHI, chalcone isomerase; F3H, flavanone-3-hydroxylase; F3′H, flavanone-3′-hydroxylase; F3′5′H, flavanone-3′5′-hydroxylase; ANS, anthocyanin synthase; FLS, Flavonol synthase; DFR, dihydroxyflavone reductase; UFGT, flavonoid-3-o-glucosyltransferase; MYB113, MYB transcription factor 113; PAP1, production of anthocyanin pigment 1, MYB75; PAP2, production of anthocyanin pigment 2, MYB90; TT8, transparent testa 8; EGL3, enhancer of glabra; and TTG1, transparent testa glabra 1. ABTS (2, 2′-azino-bis(3-ethylbenzothiazoline-6-sulfonic acid))” and “DPPH (1,1-Diphenyl-2-picrylhydrazyl radical 2,2-Diphenyl-1-(2,4,6-trinitrophenyl)hydrazyl).*
