# Supplementary material for: Global Transcriptome Analysis Revealed the Molecular Regulation Mechanism of Pigment and Reactive Oxygen Species Metabolism During the Stigma Development of Carya cathayensis
Source: Front Plant Sci. 2022 May 9;13:881394. doi: 10.3389/fpls.2022.881394 (PMC9125253; doi:10.3389/fpls.2022.881394)
Supplement: Supplementary file 1 [file Presentation_1.pptx]

## Slide 1
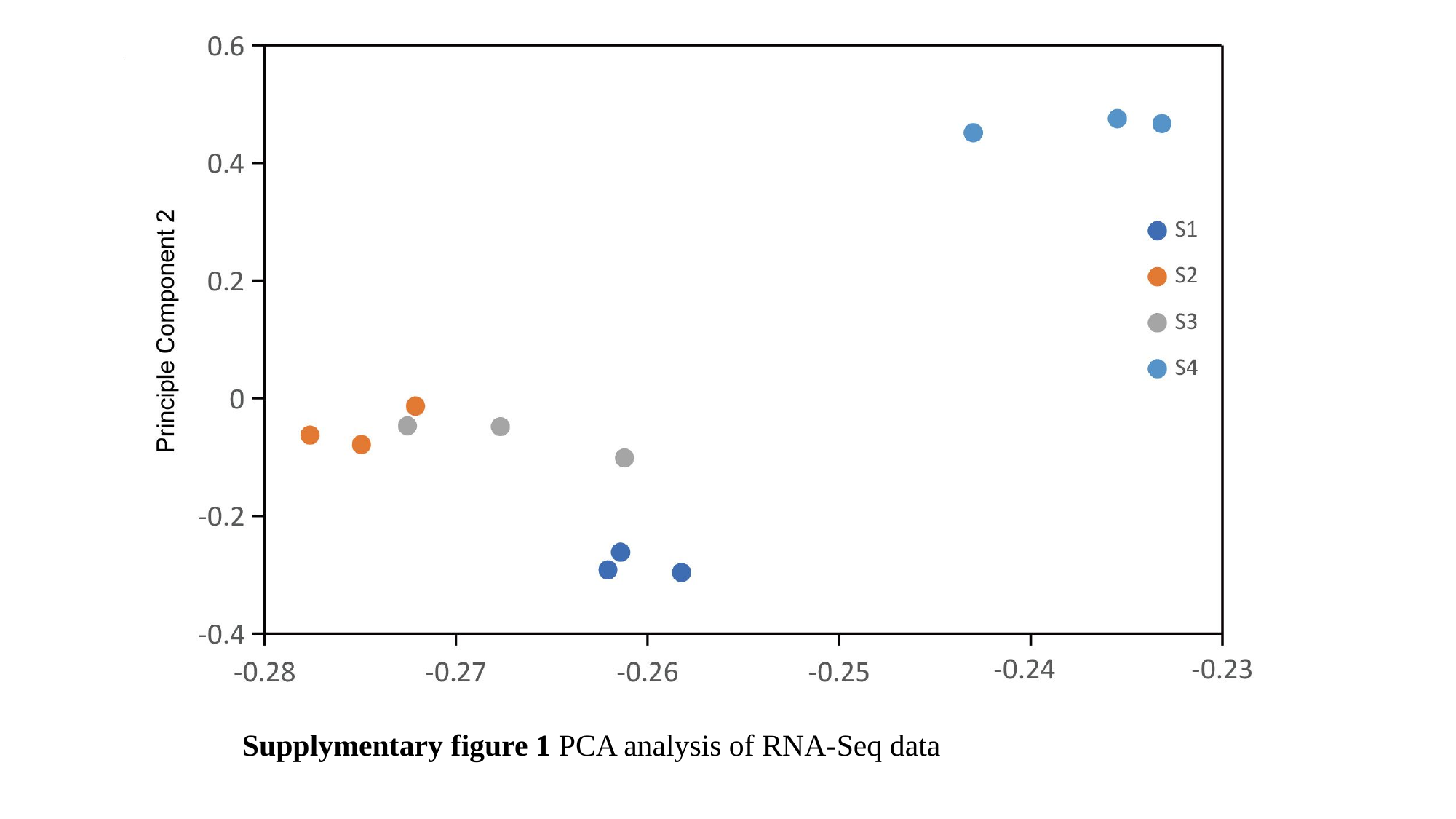

Supplymentary figure 1 PCA analysis of RNA-Seq data

## Slide 2
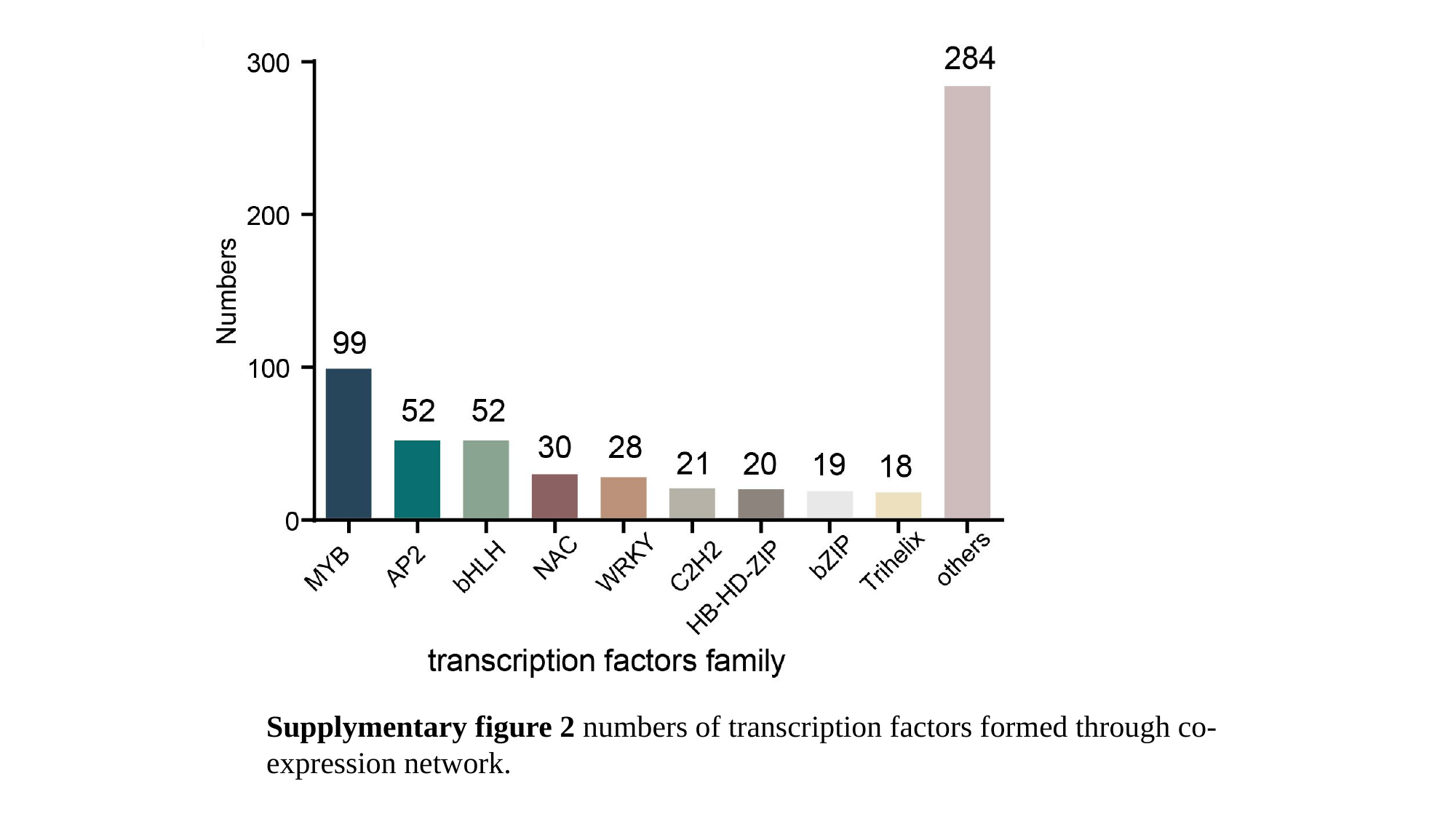

Supplymentary figure 2 numbers of transcription factors formed through co-expression network.

## Slide 3
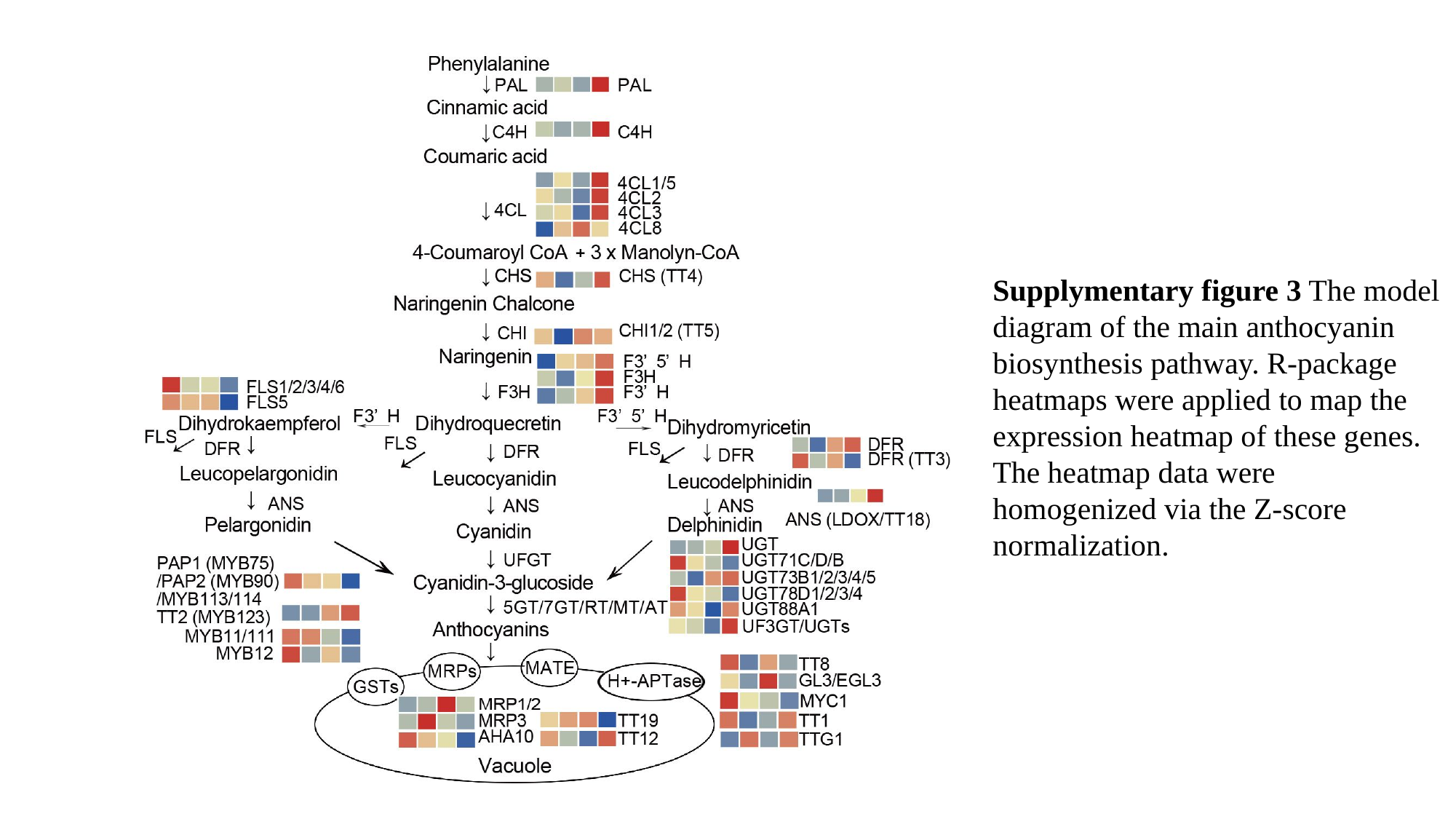

Supplymentary figure 3 The model diagram of the main anthocyanin biosynthesis pathway. R-package heatmaps were applied to map the expression heatmap of these genes. The heatmap data were homogenized via the Z-score normalization.
